# The Chicago Board of Health

**Published:** 1867

**Authors:** 


*The Chicago Board of Health* have entered upon the active discharge of the duties pertaining to their appointment. In the list of their names are to be recognized many returned army surgeons thoroughly familiar with hygienic methods, and awake to their great importance. They will see to it that this great camp of two hundred and fifty thousand "battling for life" is thoroughly policed.

As an earnest of what is to be done we give in the JOURNAL this prominent position to "Circular No. 1" and its appended paper. We shall be "amang them, takin' notes" the ensuing months.

[*Circular No. 1.*]

OFFICE OF SANITARY SUPERINTENDENT,
BOARD OF HEALTH,
*Chicago, April 18th, 1867.*

The Sanitary Inspectors will keep constantly in mind, the great good to society and the scientific value of the knowledge expected to be gained by the system of Health Police that has just been inaugurated in this City. It is therefore desirable that they should take special interest in all sanitary questions and keep themselves informed of what is being done at home and abroad relative to the causes which affect health or disease; thus they will contribute their share to the accumulation of knowledge which is destined to promote human life and establish sanitary science on the most permanent foundations. The value of these inspections depend entirely upon the conscientiousness, intelligence and industry of the Inspectors, which the

Board trusts will be appreciated, particularly when it is borne in mind that they involve life, health, and property.

Inspectors will be under the immediate control of the Superintendent, and will obey orders with promptness. They shall present themselves at the office of the Sanitary Superintendent as often as required by him, to receive orders and make reports; nor are they to consider that their duties are limited to the district to which they may be assigned. They are to watch over all cases of small-pox, malignant fevers, or any contagious and infectious diseases, and if the patients are removed, follow them with their supervision. It is a matter of great importance that they should familiarize themselves without delay with the sanitary condition of their respective districts, commencing their inspections in the sections of it where sanitary reform is most needed, and devoting special attention to the localities in which cholera prevailed during the past summer, diligently searching for all local causes of disease, especially in over-crowding, in the lack of proper ventilation, drainage, and water, (and the influence of manure, garbage, &c., in such common use for filling up lots in this city,) and if possible to indicate in their reports the remedy for such deficiencies when found. The Inspectors shall every Saturday make a written report to the Superintendent, stating what duties he has performed during the past week and also such facts as may have come to his knowledge connected with the sanitary affairs of his district or the city, worthy of the attention of the Board, or as its regulations may require. All the forms for making reports must be filled up legibly and minutely, and any information added that will throw light on the subject under investigation. Reports should contain the name and position of the person making it, the date when any matter was inspected, the streets, avenues, and alleys, if any, between; how many lots the same is upon, the names of the several owners, tenants, and occupants of each, so far as ascertainable, reports should specify what part of the thing reported upon is on each lot, and (except in case of the regular general reports of Inspectors, and upon which no order of the Board is to be founded,) there must be a separate report on a separate blank for each thing and lot reported upon, except, that when one building or business owned, tenanted, or occupied by the same person covers several lots, only one report need be made; and in cases where it will facilitate the understanding of the thing complained of a simple diagram of the premises should be sketched with a pen on the margin of the report.

Care must be taken to secure accuracy in reporting owners, tenants, or occupants. In regard to each and every nuisance reported upon, the officer making the report should state his opinion that it is dangerous to life and detrimental to health.

In addition to the duties already imposed upon the Inspectors, they will promptly investigate and report upon any special complaints which may be referred to them by the Superintendent, and also pay particular attention to the character of meats and other articles of food offered for sale in their respective districts. They shall wear their badges prominently displayed when engaged in their official duties. On entering any house or premises, they must announce their authority and the object of their visit, and while endeavoring to avoid giving offence, must make their investigations minutely. If resistance is offered to the performance of their duty, they are to report the fact to the Superintendent. They will, likewise, promptly report all who violate the health laws, in order that the offender may be summarily dealt with. All questions of doubtful authority must be referred to the Superintendent for decision.

By order of the Board.

To Dr.--------------------------, JOHN H. RAUCH,
*Sanitary Inspector.* *Sanitary Supt.*

METHOD OF MAKING SANITARY SURVEY TO BE FOLLOWED BY INSPECTORS IN THEIR RESPECTIVE DISTRICTS.

1. Examine each square thoroughly before proceeding to the next in succession in the same range; complete the inspection of all the square comprised between two contiguous parallel streets from one extremity of the district to the other, returning upon the range of square parallel and adjacent to the first to the beginning of the district; to start again upon the third range and return on the fourth, as before; and so proceed to the end.

2. The examinations should be made, as far as necessary, from house to house, and should be thorough.

3. The leading subjects of inquiry are comprised under the following heads, with such amplifications as the experience of the inspector or circumstances may suggest:

*Section I.*

1. No. of district.
2. Boundaries of district.

3. Superficial area.
4. No. and names of streets and parts of do. and alleys.
5. Superficial area of streets and parts of do. and alleys.
6. No. of blocks.
7. Superficial area of blocks.
   *a*—Superficial area of blocks covered by buildings.
   *b*—Superficial area of blocks, yards, and courts.
   *c*—Superficial area of blocks, and vacant lots.

*Section II.—Grade and Pavement.*

1. Maximum height above Lake level.
2. Minimum height above Lake level.
3. Depth to clay.
4. Character of material used in raising the grade.
5. Streets raised to grade, with area of same.
6. Streets partially raised to grade, with area of same.
7. Streets below grade, with area of same.
8. Lots raised to grade, with area of same.
9. Lots not raised to grade, with area of same.

*Section III.—Drainage.*

1. Natural drainage.
2. Artificial drainage.
   *a*—No., size, and extent of public sewers, and condition.
   *b*—No., size, and extent of private sewers, and condition.
   *c*—No., size, and extent of lots drained.
   *d*—No., size, and extent of lots undrained.
   *e*—No. of houses with sewer connections.
   *f*—No. of houses with sewer connections, together with character of same, whether perfect, imperfect, or bad.

*Section IV.—Water Supply.*

1. No. of lots supplied.
2. No. of lots unsupplied.
3. No. of street hydrants and fire plugs, and distance of same apart.

10. Area of paved streets and alleys, with No., and name, and character of pavement.

11. Area of sidewalks, with No., name, and character of pavement.

*Section V.—Buildings.*

1. No and character of buildings.
   *a*—Brick.
   *b*—Wood.
   *c*—Dwelling houses, and hotels.
   *d*—Stores, factories, workshops, etc.
   *e*—Mixed.
   *f*—Saloons, brothels, etc.
2. Basements, and their condition.
3. Ventilation of buildings.
   *a*—Heating and lighting same.
4. Location and character of water closets and privies.

*Section VI.—Population.*

1. Prevailing character of, and No. of each class, viz.: males, females, white, and colored.
3. Nationalities.

*Section VII—Hygiene.*

1. Present hygienic condition, and cleanliness.
2. Sources of dirt accumulations.
3. Diseases prevailing during past year, with locality of same.
4. Probable causes of same.
5. Relative mortality.
6. Vaccination. How many.
   *a*—How many twice.
   *b*—How many never.

